# An HR-MAS MR Metabolomics Study on Breast Tissues Obtained with Core Needle Biopsy

**DOI:** 10.1371/journal.pone.0025563

**Published:** 2011-10-18

**Authors:** MuLan Li, Yonghyun Song, Nariya Cho, Jung Min Chang, Hye Ryoung Koo, Ann Yi, Hyeonjin Kim, Sunghyouk Park, Woo Kyung Moon

**Affiliations:** 1 Department of Radiology, College of Medicine, Seoul National University, Jongno-gu, Seoul, Korea; 2 Department of Biochemistry and Center for Advanced Medical Education, College of Medicine, Inha University, Chung-gu, Incheon, Korea; Instituto de Investigación Sanitaria INCLIVA, Spain

## Abstract

**Background:**

Much research has been devoted to the development of new breast cancer diagnostic measures, including those involving high-resolution magic angle spinning (HR-MAS) magnetic resonance (MR) spectroscopic techniques. Previous HR-MAS MR results have been obtained from post-surgery samples, which limits their direct clinical applicability.

**Methodology/Principal Findings:**

In the present study, we performed HR-MAS MR spectroscopic studies on 31 breast tissue samples (13 cancer and 18 non-cancer) obtained by percutaneous core needle biopsy. We showed that cancer and non-cancer samples can be discriminated very well with Orthogonal Projections to Latent Structure-Discriminant Analysis (OPLS-DA) multivariate model on the MR spectra. A subsequent blind test showed 69% sensitivity and 94% specificity in the prediction of the cancer status. A spectral analysis showed that in cancer cells, taurine- and choline-containing compounds are elevated. Our approach, additionally, could predict the progesterone receptor statuses of the cancer patients.

**Conclusions/Significance:**

HR-MAS MR metabolomics on intact breast tissues obtained by core needle biopsy may have a potential to be used as a complement to the current diagnostic and prognostic measures for breast cancers.

## Introduction

Magnetic resonance (MR) spectroscopic techniques has been a primary method employed in investigations of metabolite changes in biofluids such as urine, blood, and bile [1], [2], [3]. Recent technological advances have enabled detection of metabolites also in intact tissues, using magic angle spinning (MAS) methods [4]. MAS narrows the line widths of metabolite signals by eliminating dipolar relaxation in the semi-solid tissues through rapid sample spinning (typically> = 2000 Hz) at a magic angle (54.7 degrees) against the magnetic field. The resulting spectra show features with high resolution (HR) that are typically seen in solution MR data [4]. Such HR-MAS MR spectroscopy has been applied in metabolomics studies on breast, prostate, liver, colon, and lung tissues [5], [6], [7], [8], [9], [10]. In the case of breast cancer tissues, several studies employing HR-MAS MR have addressed issues including metabolite identification, diagnostic usefulness, and prognostic marker correlation [11], [12], [13], [14]. However, these studies were conducted retrospectively with surgically obtained tissues; their results, therefore could not be directly applicable to surgical decision making or to cancer patients that do not need axillary dissection. Samples alternatively obtained by minimally invasive fine needle aspiration biopsy (FNAB) or core needle biopsy before surgery would, in fact, be applicable. A standard high-resolution MR (non-HR-MAS) spectroscopic study with FNAB has been conducted, but it, too, used intraoperative samples [15]. Moreover, this method would be ineffective with breast tissue samples obtained by core needle biopsy, due to the high lipid contents of those tissues.

The data obtained via MR spectroscopic techniques are inherently complex, and contain information on many metabolites; such data, accordingly, have been analyzed by multivariate analysis. Variables are reduced in number, and marker signals are identified by the weights of the original variables in the reduced variables that contribute to the differentiation of the classes of interest. Principal component analysis (PCA), partial least square-discriminate analysis (PLS-DA), and neural networks are among the frequently-used methods for breast cancer metabolomics studies [12], [16]. Recently, Orthogonal Projections to Latent Structure-Discriminant Analysis (OPLS-DA) was proposed as an effective tool for metabolomic analysis [17], [18]. The main merit of OPLS-DA is its separation of the class-orthogonal variations that can obscure class differentiation. It is similar to the combination of orthogonal signal correction and PLS-DA, but, advantageously, can be completed in a single analysis. Its utility in fact has been shown in many metabolomics studies in which intra-group variation is very large [1], [19], [20].

We prospectively conducted HR-MAS MR spectroscopic studies on breast tissue samples obtained by percutaneous core needle biopsy. We employed CPMG pulse sequence which can selectively suppress signals with short relaxation times, most notably, lipid signals abundant in core needle biopsy samples. An OPLS-DA analysis yielded information on elevated metabolites in the cancer samples as well as quantitative measures on the performance of our approach in classification and blind sample prediction. On the basis of the results, we believe that minimally invasive core needle biopsy combined with the HR-MAS MR metabolomics approach may complement the currently existing breast cancer diagnostic and assessment measures.

## Results

### HR-MAS MR spectra of core needle biopsy samples

**Figure 1:**
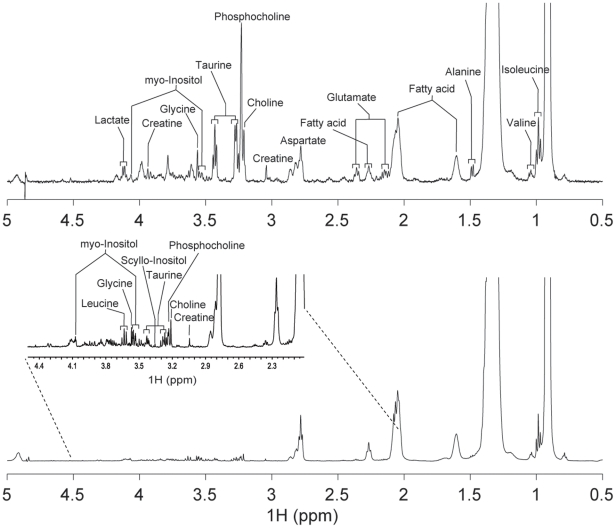
HR-MAS MR spectra of breast tissue samples obtained by 14-gauge core needle biopsy. Representative 500 MHz HR-MAS MR spectra of breast samples from a cancer patient (upper) and a non-cancer patient (lower). The spectra were taken for an average of 12.2 mg of core needle biopsy samples in D_2_O and 0.01% TSP with CPMG pulse sequence and 2 KHz spinning. Individual choline species were identified according to previous reports [13], [36] and comparison with an authentic choline sample.

The 31 breast tissue samples (13 cancer, 9 benign and 9 normal) obtained by core needle biopsy were examined, and the representative MR spectra of cancer and non-cancer samples are shown in Fig. 1. As has been the case with spectra previously reported for samples obtained through surgery, our MR spectra featured large peaks at 0.91 and 1.31 ppm due to the aliphatic fatty acid sidechains of lipids. These peaks were by far the most intense, even with the CPMG T2 filter and regardless of the cancer status, indicating that it is not easy to avoid inclusion of adipose tissues in core biopsy samples. The intensity variations of those signals were so large as to dwarf those of any others. Therefore, we excluded them from the subsequent analysis. Still, there were readily observable and reasonably resolved signals in the 2.2∼4.2 ppm region. In addition, the S/N ratios were adequate for identification of a number of metabolites that have been reported in surgically obtained samples (Fig. 1).

### Multivariate analysis of MR spectral data

**Figure 2:**
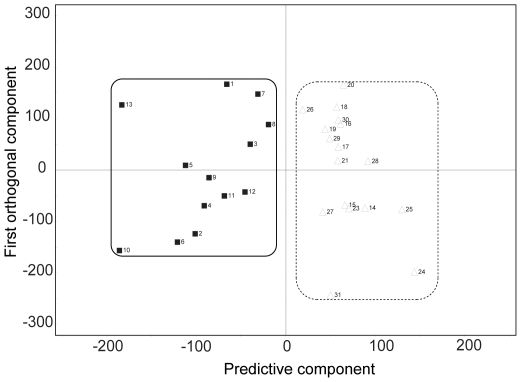
Multivariate discrimination model for cancer and non-cancer samples. Orthogonal Projections to Latent Structure-Discriminant Analysis (OPLS-DA) score plot for cancer and non-cancer samples. The model was obtained using one predictive and two orthogonal components. Filled box and solid line: cancer samples; open triangle and dotted line: non-caner samples.

As it was difficult to isolate differences between the patient groups by simple visual inspection due to the large intra-group variation, we performed a multivariate statistical analysis for a more holistic view of the data. We used the 0.99∼5.59 ppm region, but excluded water and aliphatic fatty acid sidechains signals, as stated above. Initially, we wanted to see if the approach could discriminate among the three groups (cancer, benign tumor, and normal), but found that it was not possible to distinguish differences within the non-cancerous samples (benign tumor vs. normal; data not shown). Therefore, we tried to build a model that can address the difference between cancer and non-cancer groups using the OPLS-DA approach, by which structured noise can be dealt with efficiently [1], [18], [21]. The resultant OPLS-DA model, for all of the 31 samples, separated the two major groups, cancer (n = 13) and non-cancer (n = 18), without overlap using one predictive and two orthogonal components (Fig. 2). Overall, though each sample within a group showed considerable variation, our model could discriminate them very effectively.

**Figure 3:**
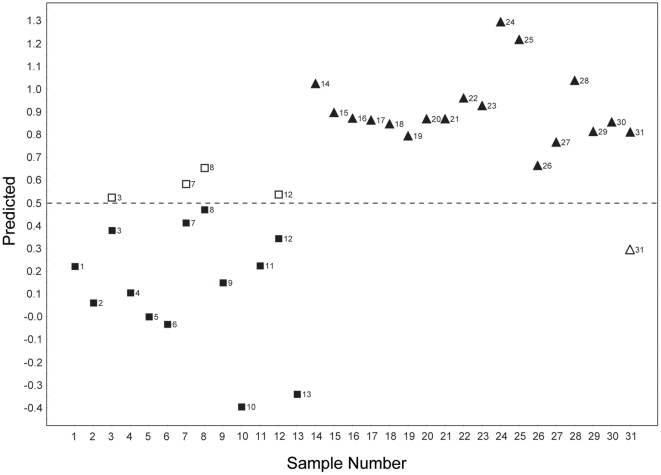
Prediction result for cancer status based on OPLS-DA model. One patient sample was left out at a time, and a new OPLS-DA prediction model was constructed with the rest of the data. The class membership of the left-out samples was predicted using an *a priori* cutoff value of 0.5 (dashed line). Filled box: cancer samples, filled triangle: non-cancer samples. The Y values of the filled symbols are from the analysis using the entire dataset. In the case of mis-classified samples, the predicted Y values are also shown as open boxes (cancer samples) and open triangles (non-cancer samples).

Although a perfect separation was achieved (see the Fig. 2: score plot), it was yet possible that the distinction was due to model over-fitting. Therefore, we performed a predictive test by leaving out one patient sample at a time and constructing the OPLS-DA prediction model with the rest of the data. The cancer or non-cancer status of the left-out sample was then predicted based on the new model. This step amounts, then, to a blind test for an unknown sample, and as such can serve as a cross-validation for the distinction model. The prediction approach was taken with the same number of predictive and orthogonal components as in the original OPLS-DA model. The class membership of the left-out sample was predicted using an *a priori* cut-off value of 0.5. The prediction results showed that the model correctly predicted 26 samples out of the total 31 (Fig. 3). Among the incorrectly predicted samples were four cancer samples predicted as non-cancer samples, and one non-cancer sample predicted as a cancer sample. Thus, the sensitivity, specificity, and accuracy were 69% (9/13), 94% (17/18), and 84% (26/31), respectively, in the prediction of the cancer status.

### Analysis of group-relevant signals

**Figure 4:**
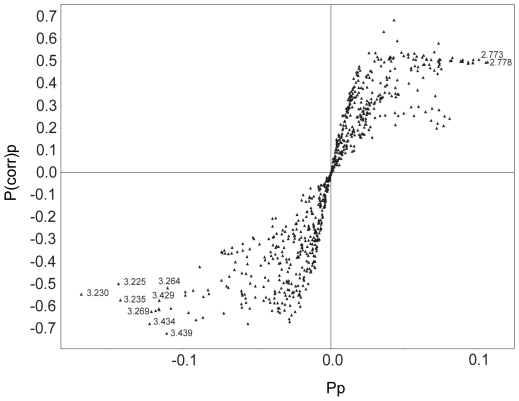
Signals contributing to differentiation. Signals contributing to the differentiation of cancer and non-cancer samples are plotted based on their *p*(corr)_p_ and *p*_p_ values. These values represent modeled correlation and modeled covariation, respectively. The most relevant chemical shift values are shown next to the symbols representing the signals.

**Figure 5:**
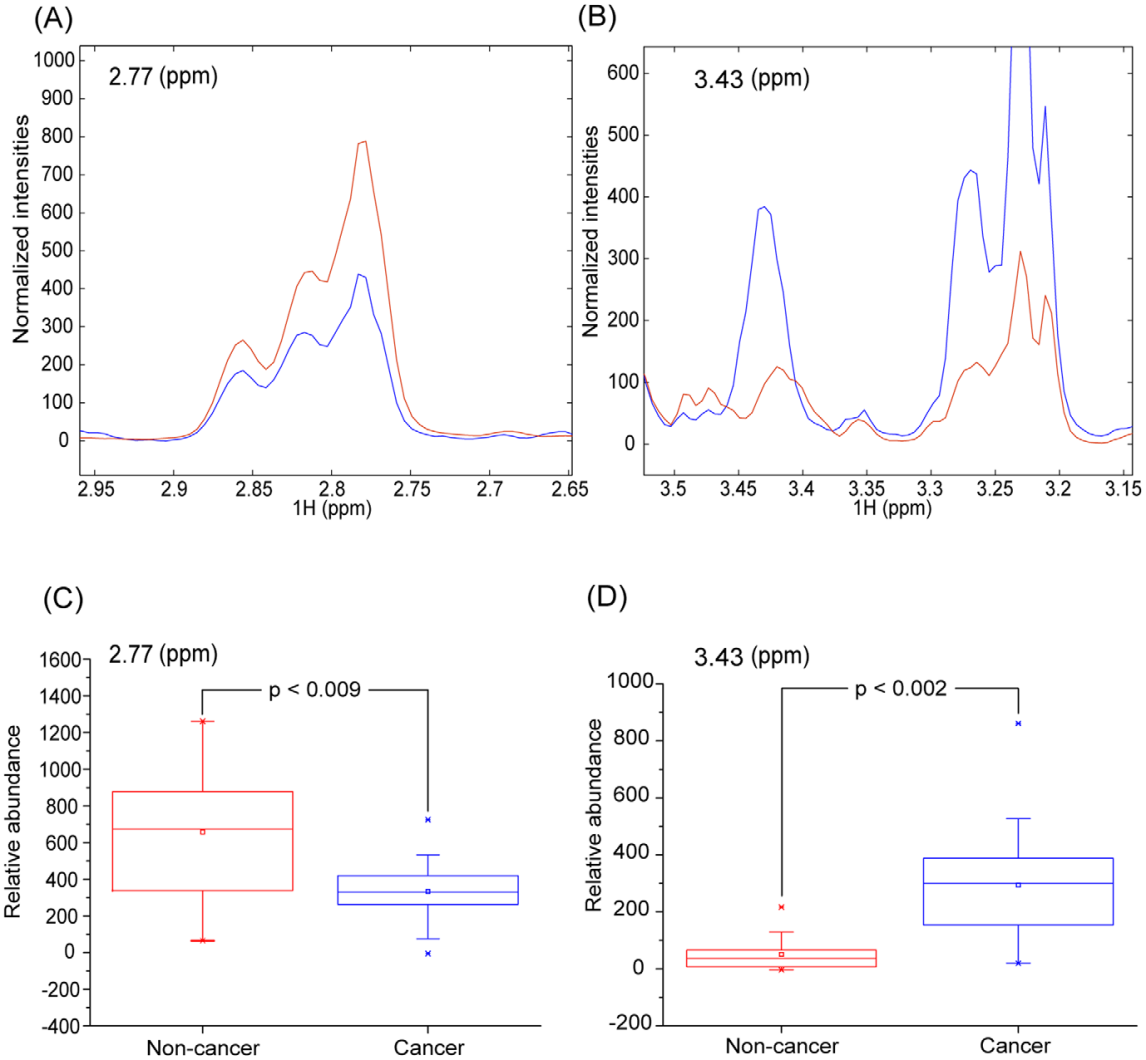
Average spectra and Mann-Whitney U test for marker signals. The levels of the makers identified by the multivariate analysis were assessed by average spectral plot and Mann-Whitney U-test. A and B: Normalized and averaged intensities of the indicated marker signals from the cancer and non-cancer samples. C and D: Box plots of the Mann-Whitney U test results with the resulting p values. In all of the plots, red represents the non-cancer samples and blue the cancer samples.

After the establishment of the model, we tried to identify the variables responsible for the differentiation of the cancer and non-cancer groups. We built an S-plot that shows the modeled correlation (*p*(corr)_p_) and covariation (*p*_p_) in a single figure, enabling easy selection of significant markers among noisy signals. The *p*(corr)_p_ values of the signals suggest that multiple signals account for group differentiation (Fig. 4) [1], [2]. Still, we could pick up 3.43 and 2.77 ppm signals as the most reliable contributors for the cancer and non-cancer groups, respectively, as they had large values for both correlation and covariation. Based on the above signal assignments, the signals were identified as coming from taurine (3.43 and 3.26 ppm) and aspartate (2.77 ppm). The assignment of aspartate was tentative, though, as its signal was broad and possibly overlapped with those from other metabolites. The signals from choline-containing compounds (3.22∼3.24 ppm), particularly phosphocholine centered at 3.230 ppm, were also correlated with the cancer group. To test the statistical validity of the signals found by this multivariate analysis, we carried out a Mann-Whitney U-test (Fig. 5) [22]. In addition, we obtained the average spectra of each group after normalization and alignment (Fig. 5). Both of these analyses showed that taurine and aspartate had a biased distribution in the cancer and non-cancer groups, respectively.

### Correlation with prognostic markers

**Figure 6:**
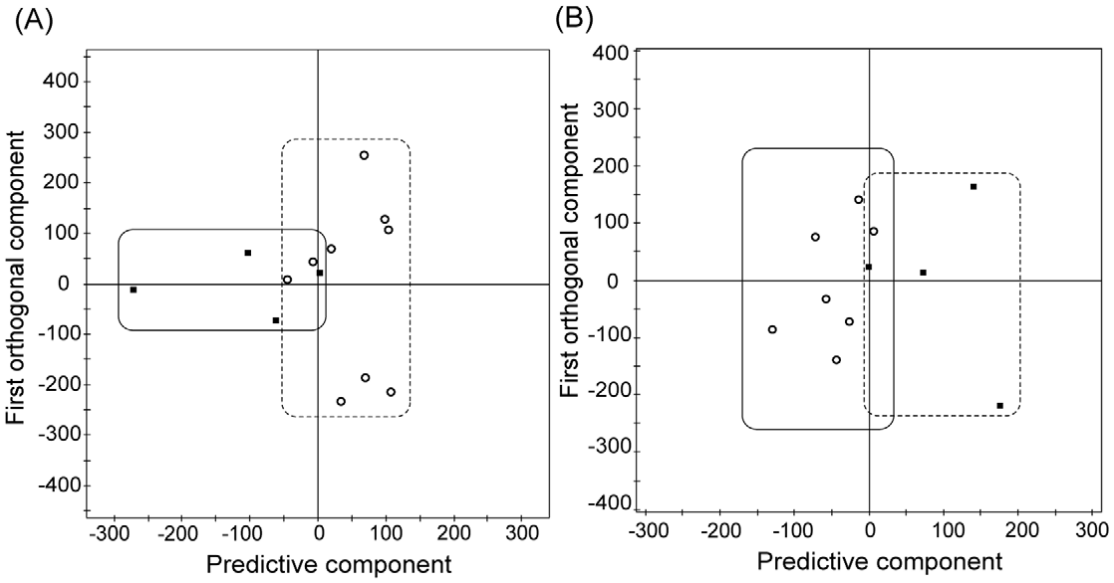
Discrimination based on prognostic markers: PR and lymph node metastases. OPLS-DA score plots based on the statuses of progesterone receptor (A) and lymph node metastasis (B). All of the models were obtained using one predictive and two orthogonal components. Two samples were excluded based on the PCA analysis for (B). Filled box: positive samples, Open circle: negative samples.

Based on the cancer/non-cancer correlation with the MR spectral data, we tested if PR status, an important prognostic marker, can also be correlated. We divided the cancer patient group into two according to the PR status (positive or negative), and obtained an OPLS-DA separation model of the MR data of each group (Fig. 6A). Although we observed cross-over of some samples along the *p*_p_ line of the model, we could see general clustering of the samples into their respective regions. We also tested the predictability of the model on blind samples using the same method used for cancer/non-cancer status. Out of the total of 13 cancer samples, 10 were predicted correctly and 3 were mis-predicted, with 1 PR-positive and 2 PR-negative samples among the latter. Other important prognostic markers, ER status and HER-2/neu, could not be evaluated, due to the small number of patients with ER negative (n = 2) and HER-2/neu negative (n = 1) in our cancer patient group (See Table 1).

**Table 1:** Tumor characteristics of the 13 patients with breast cancer.

Characteristics	Summary (n)
Tumor size	
Mean (sd)	1.4±1.6 cm
Range	0.5–3.5 cm
Histologic type	
Ductal carcinoma in situ	2
Invasive cancer	11
Low grade	2
Intermediate Grade	6
High Grade	3
Lymph node metastases	
No	9
Yes	4
Receptor and HER-2/neu status	
ER+ PR+ HER-2/neu+	6
ER+ PR− HER-2/neu+	4
ER+ PR− HER-2/neu−	1
ER− PR− HER-2/neu+	2

We also evaluated the correlation of MR spectral data with the axillary lymph node metastasis status. Inclusion of all of the cancer patients (metastasis = 4, non-metastasis = 9) did not yield a reliable discrimination model. Exclusion of two possible outliers (both from the non-metastasis group) based on a PCA and subsequent OPLS-DA modeling resulted in a reasonable distinction between the two groups (Fig. 6B). Three of the four samples in the axillary lymph node metastatic group could be separated from seven samples in the non-metastatic group.

## Discussion

We evaluated the relevance of HR-MAS MR metabolomics to core needle biopsy samples in breast cancer diagnostics. Although there have been studies using similar spectroscopic techniques on surgically obtained breast cancer samples, there have been none, to our knowledge, that have utilized intact breast tissue obtained by 14-gauge core needle biopsy. As percutaneous image-guided biopsy using an 8–14 gauge needle is a standard procedure for inspecting suspicious breast lesions in most hospitals, the results can be directly translated into real clinical situation. Another important advantage of our approach is that HR-MAS MR spectroscopy is non-destructive, enabling re-use of samples for later histopathological examinations [14], [16]. We did not find any degradation of the tissue samples after HR-MAS MR spectroscopy and successfully performed H&E staining and immunohistochemistry analysis. One possible caveat regarding the core biopsy approach is the small amount of the obtained sample and uncertainties associated with the actual sampling positions for small tumors. Still, it seems that the metabolomics approach might be more suited to analyzing core-biopsy samples than other-omics approaches. This is due to the fact that the small molecules analyzed by MR-based metabolomics are more diffusible than proteins or DNA molecules, and, therefore, can reflect the status of neighboring tissues better than much larger macromolecules. Again, the non-destructive nature of the technique is in contrast with proteomics and genomics, which entail sample destruction. The current study used normal and benign tissue samples for comparison with cancer samples, unlike previous studies, which used non-involved tissues from cancer patients [11], [15], [16], [23]. In addition, these earlier studies included relatively advanced-cancer patients undergoing surgery, which fact might have facilitated the tissue distinction. Therefore, our patient group might be more variable even though the sample size is much smaller than some of the previous ones.

Mountford et al. reported the diagnostic utility of *ex vivo* HR-MR spectroscopy combined with linear-discriminant analysis (LDA) for FNAB samples from breast cancer patients [15]. They tried the same approach for core needle biopsy samples, but were unsuccessful due to the high fat content masking diagnostic signals [15]. The successful discrimination of cancer status with core needle biopsy samples in our present study was owed to several methodological differences. First, we used HR-MAS, which can significantly narrow the line width of signals from semi-solid tissue samples. This line-narrowing is directly translated into increased signal intensities. Second, we also employed CPMG pulse sequence, which can selectively suppress signals with short relaxation times, most notably, lipid signals. This increases the relative contributions of other regions that carry diagnostic information. Third, despite the use of the above spectroscopic techniques, saturated fatty acid signals were the most intense peaks. Therefore, we removed those regions from the spectra and normalized the data with the total integral of the remaining regions. This step proved to be particularly important, as a variety of other normalizations failed to produce acceptable results. In addition to the above measures to obtain or process the spectral data, the use of OPLS-DA multivariate analysis [17], [18] also contributed to our results. OPLS-DA is different from PLS-DA in that it rotates the score matrix so that the class-orthogonal variation can be separated from the class-predictive one. Therefore, it can provide easier interpretation of the factors contributing to class difference in the presence of large intra-group variation, such as that seen in the current case. HR-MAS MR spectroscopy with CPMG pulse sequence has been applied to tissue samples obtained during surgical procedures [11], [12], [14], [23]. However, adipose tissues could be physically avoided in those cases [14], whereas, in core needle biopsy, their contents cannot be controlled, and the resulting samples may be difficult to analyze with more conventional approaches. OPLS-DA has been also used successfully to analyze other demanding metabolomic cases [1], [20], [24], including the one concerning data obtained from genetically homogenous animals [25].

In addition to the above stated merits, OPLS-DA provided easily interpretable data (S-plot, see Fig. 4) concerning metabolites relevant to discrimination of cancer and non-cancer groups. Our data showed that taurine and choline-containing compounds, especially phosphocholine, were elevated in the cancer samples compared with the non-cancer ones. Choline-containing compounds have been found to be elevated in breast cancer [26], [27], [28], as well as in other malignancies [29]. In addition, phosphocholine level was higher in breast cancers or cancer cell lines than normal counterparts [30], [31], [32]. Taurine levels have also been known to be higher in prostate [33] and breast cancers [34]. This consistency supports the relevance of our approach using minimally invasive HR-MAS MR spectroscopy with core needle biopsy in metabolite analysis of cancer tissues. There have been several studies on the diagnostic performance of MR spectroscopic techniques with biopsy-obtained breast cancer samples. In one such report, HR-MR spectroscopic data on intraoperative FNAB samples analyzed by multivariate analysis showed 94% sensitivity and 98% specificity in discriminating cancer and non-cancer tissues [15]. Another study using HR-MAS MR spectroscopy on surgically obtained tissue samples reported 82% sensitivity and 100% specificity based on the intensities of choline-containing compounds [13]. It should be noted that the values of the former study were obtained with only a training set, and those of the latter were from an intensity comparison of choline that is not applicable to multivariate blind tests. If we apply the same criterion, that is, diagnostic performance on a training set without a blind test, to the OPLS-DA classification model, we obtain 100% for both sensitivity and specificity based on the predictive component. However, these approaches tend to yield over-optimistic values, and more relevant estimation should be obtained with blind tests using samples that were not used to build the classification model [12]. It is notable that blind sample prediction was done to evaluate the performance of prognostic markers from surgical samples in later studies [12], [16]. A blind test on our data set, excluding one sample at a time until all of them had been left out showed 69% sensitivity, 94% specificity and 84% accuracy. Another intriguing feature of our results is the correct blind-test prediction of ductal carcinoma in situ, a very early stage cancer. Although the number of cases was small (n = 2), this could be an interesting point to focus on in a larger study.

Our approach did not yield a reliable discrimination model for ER status (data not shown), whereas it did provide a reasonable distinction for PR status. Recently, Giskeødegård et al. presented a good prediction for the two prognostic markers based on HR-MAS studies with surgically obtained tissue samples [12]. In their case, most patients had a similar ER/PR status whereas the two factors were hardly correlated in our patient groups. Therefore, it is not surprising that there are differences between the results for their and our patient groups. Another important prognostic marker is axillary lymph node status. We could obtain a reasonable classification only after the exclusion of two non-metastatic samples based on a PCA analysis. At this point, it is not clear what properties of these two samples made them closer to the metastatic ones, which issue might be elucidated with a larger-sample-size study. Still, the difficulty in lymph node status prediction is not surprising in that a recent study also reported an unsatisfactory result [12]. As noted in that study, earlier high-accuracy results [15] could not be directly compared, due to the lack of a blind test.

In conclusion, the HR-MAS MR metabolomics approach was shown to be feasible with intact breast tissues obtained by core needle biopsy. Specifically, our results show that this approach has the potential to discriminate cancer and non-cancer and to classify breast cancers according to their metabolite profiles. If validated in a larger study, the approach may be used as a complement to the current diagnostic and prognostic measures for the management of women with breast cancers.

## Materials and Methods

### Ethics Statement

Institutional review board approval was obtained for this prospective study from the Seoul National University Hospital Institutional Review Board (H-1003-037-312), and all patients provided written informed consent.

### Patients

Between May 2010 and November 2010, 22 consecutive women (mean age, 49 years; age range, 20–68 years) who had been scheduled to undergo an ultrasound-guided percutaneous 14 gauge- core needle biopsy were examined. We had obtained the 31 breast tissue samples (13 cancer, 9 benign and 9 normal) from a total of 22 women (13 patients with breast cancer and 9 patients with benign tumors) (Table 2). The normal breast tissue samples were obtained from sites adjacent to the periphery of the benign tumors from patients with benign breast tumors by simply changing the direction of the needle. Among the cancer patients, eleven of them had infiltrating ductal carcinoma and the rest of two had ductal carcinoma in situ. Four of the patients with infiltrating ductal carcinoma also had metastasis on the lymph nodes (Table 1). We did not apply any exclusion criteria and analyzed all the samples of the patients enrolled in the study to maximize the patient diversity.

**Table 2:** Clinical and histological data on 31 samples from 22 patients included in the main study.

Characteristics	Summary (n)
Age (years)	
Median	50
Mean (range)	49 (20–68)
Histologic Diagnosis	
Cancer	
Infiltrating Duct Carcinoma (IDC)	11
Duct Carcinoma In Situ (DCIS)	2
Benign	
Fibroadenoma	6
Fibrocystic change	2
Adenosis	1
Normal*	9

*Normal samples (n = 9) were obtained from the patient with benign tumors.

For HR-MAS MR spectroscopy, tissue samples were placed in cryogenic vials and were immersed in liquid nitrogen immediately after dissection.

### MR spectroscopy data acquisition

All one-dimensional HR-MAS MR spectra of the tissue samples were measured with an NMR spectrometer (Agilent, VNMRS 500) operating at a proton NMR frequency of 500.13 MHz (11.7 T). Temperature was set to 19°C after calibration with methanol. Each experiment took 1 hour and 5 minutes.

Frozen samples were thawed in NMR laboratory, weighed, and placed into an HR-MAS nano-probe® (Agilent, Walnut Creek, CA). The total volume of the sample cell is 40 µl, and an average of 12.2 mg core-biopsy samples were put in the cell with the remaining volume filled with D_2_O (0.01% TSP). The probe was an inverse-detection type and equipped with single Z-gradient coil. The spectra were taken with CPMG pulse sequence to impose a T2 filter. The total T2 delay was set to 290 msec and the sample was spun at 2 KHz. The spectra were acquired with total complex points of 16 K, sweep width of 7961 Hz, and 1024 transients. The 90 degree pulse was calibrated with each sample on water resonance. Water signal was saturated using weak power continuous wave during the recycle delay.

### Data processing

The time-domain spectra were apodized with exponential function (1 Hz), and then Fourier-transformed, phased and baseline-corrected manually. Spectra were referenced to the TSP signal at 0.00 ppm which was also checked by alanine signals at 1.48 ppm in case the TSP signal is split due to protein binding. To reduce the complexity of the NMR data for the subsequent multivariate analysis, the spectra were binned by 0.005 ppm interval and normalized by integration values over the region of 0.99∼5.59 ppm. As the aliphatic lipid signals were vastly different from sample to sample, only the regions that are not affected by those signals were used (1.44∼1.91 ppm and 2.15∼5.59 ppm). Within those regions, the water region (4.61∼5.03) was excluded in the normalization due to its irregular behavior. These binning and normalization were done using an in-house built Perl program. To compensate for possible peak shift mismatch due to the relatively high resolution binning, the spectra were aligned using correlation-optimized warping algorithm [35].

### Multivariate and spectral analysis

Matlab (MathWorks, Natick, MA), SIMCA-P 11.0 (Umetrics, Sweden), and Excel (Microsoft, Seattle, WA) programs were used to process the numeric data for statistical analysis. Chenomx (Spectral database; Edmonton, Alberta, Canada) was used for spectral analysis. Principal component analysis, partial least square-discriminant analysis, and OPLS-DA were performed to identify latent patterns and distinguish patient groups. Class discrimination models were built until the cross-validated predictability value does not meaningfully increase to avoid over-fitting of the statistical model. The statistical model was validated by prediction of the unknown samples using leave-one-out analysis. An a priori cut-off value of 0.5 was used to evaluate the prediction results [2]. Signals contributing to the class differentiation were identified by S-plot and the corresponding metabolites were identified using Chenomx (Spectral database; Edmonton, Alberta, Canada) software and an in-house built database.

### Immunohistochemistry

After HR MAS analysis, each core needle biopsy specimen was fixed in ice-cold acetone for histopathology. One 5 mm section was cut from each frozen tissue, and stained with haematoxylin-eosin (H&E) for microscopic examination by a pathologist. Another section was stained immunohistochemically for estrogen receptor (ER), progesterone receptor(PR), and HER-2/neu using monoclonal mouse-anti-human ER (ab7825, abcam, USA), PR (sc52358, santa cruz, USA) and HER-2/neu (sc71667, santa cruz, USA) and Dako REAL EnVision Peroxidase/DAB+ in a Dako Autostainer Plus.
